# Microvascular dysfunction in a murine model of Alzheimer’s disease using intravital microscopy

**DOI:** 10.3389/fnagi.2025.1482250

**Published:** 2025-02-10

**Authors:** Danielle Sidsworth, Noah Tregobov, Colin Jamieson, Jennifer Reutens-Hernandez, Joshua Yoon, Geoffrey W. Payne, Stephanie L. Sellers

**Affiliations:** ^1^Division of Medical Sciences, University of Northern British Columbia, Prince George, BC, Canada; ^2^Department of Radiology, Faculty of Medicine, University of British Columbia, Vancouver, BC, Canada; ^3^Cardiovascular Translational Laboratory, Providence Research and Centre for Heart Lung Innovation, Vancouver, BC, Canada; ^4^Biochemistry and Molecular Biology Program, University of Northern British Columbia, Prince George, BC, Canada; ^5^University of Northern British Columbia, Prince George, BC, Canada; ^6^Centre for Cardiovascular Innovation, St. Paul’s and Vancouver General Hospital, Vancouver, BC, Canada; ^7^Centre for Heart Valve Innovation, St. Paul’s Hospital, University of British Columbia, Vancouver, BC, Canada; ^8^Dilawri Cardiovascular Institute, Vancouver General Hospital, Vancouver, BC, Canada

**Keywords:** Alzheimer’s disease, microvascular dysfunction, murine model, neurodegeneration, vasoreactivity, intravital microscopy

## Abstract

Alzheimer’s disease (AD) is a complex neurocognitive disorder. Early theories of AD sought to identify a single unifying explanation underlying AD pathogenesis; however, evolving evidence suggests it is a multifactorial, systemic disease, involving multiple systems. Of note, vascular dysfunction, encompassing both cerebral and peripheral circulation, has been implicated in AD pathogenesis. This pilot study used intravital microscopy to assess differences in responsiveness of gluteal muscle arterioles between a transgenic AD mouse model (APP/PS1; Tg) and wild-type (C57BL/6; WT) mice to further elucidate the role of vascular dysfunction in AD. Arteriole diameters were measured in response to acetylcholine (10^–9^ to 10^–5^ M), phenylephrine (10^–9^ to 10^–5^ M), histamine (10^–9^ to 10^–4^ M) and compound 48/80 (10^–9^ to 10^–3^ M). Tg mice demonstrated a trend toward reduced vasodilatory response to acetylcholine with a significant difference at 10^–5^ M (36.91 vs. 69.55%: *p* = 0.0107) when compared to WT. No significant differences were observed with histamine, compound 48/80 or phenylephrine; however, a trend toward reduced vasoconstriction to phenylephrine was observed in Tg mice at higher concentrations. Mean net diameter change (resting to maximum) also differed significantly (*p* = 0.0365) between WT (19.11 μm) and Tg mice (11.13 μm). These findings suggest reduced vascular responsiveness may contribute to the systemic vascular deficits previously observed in AD models. Future research using diverse models and broader variables could further elucidate peripheral vascular dysfunction’s role in AD pathogenesis, including its impact on motor symptoms and disease progression. Such insights may inform the development of vascular-targeted therapeutic strategies.

## 1 Introduction

Alzheimer’s disease (AD) is a clinically and biologically complex neurodegenerative disorder, commonly manifesting with amnestic cognitive impairment, decline in language and executive function, and disturbances in motor function. Key risk factors of AD include age, female sex, certain genetic variants (such as apolipoprotein ε4), prior head injuries, educational background, metabolic syndrome, and lifestyle (e.g., diet, inactivity, and smoking) (Livingston et al., 2020). However, this broad and evolving array of factors, in addition to their temporal and individual variability, complicates efforts to determine the exact roles of these factors as causative agents, disease markers, or drivers of disease progression (Duchesne et al., 2024).

Historically, theories of AD pathogenesis have sought to provide a unifying etiological explanation. A 2024 scoping review by Duchesne et al. (2024), found that approximately three-quarters of 131 original manuscripts published since 1980 proposed unifactorial hypotheses, most commonly focusing on amyloidogenic, metabolic, infectious, and cerebrovascular/blood brain barrier (BBB) pathways. Among these, the amyloid cascade hypothesis (Hardy and Higgins, 1992) —which posits that abnormal amyloid precursor protein (APP) processing leads to excessive β-amyloid (Aβ) peptide accumulation, plaque formation, and subsequent neuronal dysfunction—has guided AD research and drug development for over three decades. However, this unifactorial theory has been increasingly revisited (Karran et al., 2011; Morris et al., 2014; Selkoe and Hardy, 2016) and, in part, reconsidered based on evidence that amyloid accumulation alone neither fully accounts for the clinical heterogeneity of AD (Jansen et al., 2015; Parent et al., 2023) nor guarantees that Aβ-targeted therapies will yield meaningful cognitive improvements (Asher and Priefer, 2022; Kim et al., 2022), although newer treatments display promise (van Dyck et al., 2023).

As our understanding of AD pathogenesis evolves, it is increasingly recognized that AD is not an isolated brain disorder, but rather the culmination of multifactorial, systemic, and chronic pathological processes affecting multiple organs and systems (Cerasuolo et al., 2023; Decourt et al., 2022; Duchesne et al., 2024). In this regard, the cardiovascular system has garnered particular interest (Scheffer et al., 2021). Evidence increasingly suggests that peripheral vascular health, blood flow, endothelial function, and blood-brain barrier integrity play integrals roles in AD pathogenesis (Nordestgaard et al., 2022; Scheffer et al., 2021; Venturelli et al., 2018). Vascular dysfunction and AD also appear to share common risk factors and potentially overlapping pathogenic mechanisms, such as Aβ production and vascular deposition, and endothelial damage contributing to microangiopathy (Nordestgaard et al., 2022; Venturelli et al., 2018). Notably, cerebral hemodynamic alterations appear early in AD and have been proposed as both diagnostic and prognostic markers (Saka et al., 2022), while similar disturbances in peripheral vasculature have been correlated with disease severity and may influence the course of AD through complex interactions with cerebral vessels (Venturelli et al., 2018). Despite these connections, the specific relationship between vascular disease and AD remains only partially elucidated, and how peripheral vascular dysfunction relates to cerebral changes and disease progression is not yet defined (Eisenmenger et al., 2023). Addressing these knowledge gaps and further investigating vascular contributions to AD’s heterogeneous etiology aligns with the expressed need for more integrative theoretical frameworks that capture the complexity of this condition (Duchesne et al., 2024), and such efforts have the potential to illuminate novel preventive, diagnostic, and therapeutic pathways (Barbera et al., 2023; Duchesne et al., 2024).

Intravital microscopy (IVM) is a sophisticated imaging method which allows for microscopy to be performed in living mice through a surgical window. This method employs light transmission, fluorescent dyes, conjugated antibodies, or genetically engineered *ex vivo* reporter system to system to study real-time tissue physiology (Taqueti and Jaffer, 2012). IVM can enable identification of temporal relationships between disease processes and determine cause-and-effect in responses to stimuli (Kranig et al., 2020; Stein and Gonzalez, 2017; Wissmeyer et al., 2022). Furthermore, IVM allows tissues to be examined following repeat exposures, reducing effects of biological variation between subjects, and/or experimental variation across treatments. Various IVM models have been developed to study acute and chronic vascular pathologies, such as atherosclerosis, peripheral vascular disease (PVD) and cerebral vascular dysfunction (Taqueti and Jaffer, 2012; Wissmeyer et al., 2022). This approach offers a controlled assessment of vascular function and how it relates to disease processes (Bearden et al., 2004; Kranig et al., 2020; Stein and Gonzalez, 2017; Taqueti and Jaffer, 2012; Wissmeyer et al., 2022).

Given the growing recognition that vascular dysfunction contributes to AD pathogenesis, and the identified need for multimodal preventive and therapeutic strategies (in addition to anti-amyloid treatments) (Livingston et al., 2020) exploring the relationships between peripheral vascular dysfunction and AD is necessary. In this pilot study, we investigated vasomotor reactivity in skeletal muscle of a murine model of Alzheimer’s disease (AD) using a standardized intravital microscopy (IVM) protocol. Arterioles within the gluteal muscle of APP/PS1 transgenic (Tg) and wild-type (WT) mice were exposed to vasodilatory, vasoconstrictive, and inflammatory agents to assess differences in vascular responsiveness between these two groups. We hypothesized that the arterioles of APP/PS1 transgenic mice would exhibit reduced vascular responsiveness compared to WT mice.

## 2 Materials and methods

The procedures described below were an extension of protocols developed previously (Bearden et al., 2004).

All procedures were approved by the Institutional Animal Care and Use Committee of the University of Northern British Columbia in accordance with the Canadian Council of Animal Care (CCAC) guidelines.

### 2.1 Ethics

The UNBC Animal Care and Use Committee approved the procedures conducted in this study (REB#: 2016-8). The study was conducted in accordance with ethics guidelines.

### 2.2 Animals

The transgenic/experiment group (referred hereon to as Tg) were male mice from the APP/PS1 (line 85) [B6;C3-Tg(APPswe,PSEN1dE9)85Dbo/Mmjax, #034829-JAX] strain sourced from Jackson Laboratories. These dual-genetically modified mice produce a combined mouse/human amyloid precursor protein (Mo/HuAPP695swe) and a mutated human presenilin 1 (PS1-dE9), both directed at CNS neurons and associated with early-onset familial Alzheimer’s disease. Co-expression of these proteins leads to accelerated production and aggregation of Aβ (Holcomb et al., 1998). The control/wildtype group (referred hereon to as WT) were C57BL/6 mice (stock #:000664) sourced from Jackson Laboratories.

### 2.3 Surgical preparation

Based on prior methods (Bearden et al., 2004), mice were anesthetized with isoflurane gas at an induction rate of 2–3% and supplemented as needed, and a maintenance rate of 0.5–1% to maintain the surgical plane. Withdrawal to toe pinch was monitored to ensure mice were sedated throughout the length of the experiment. Mice were secured prone on an acrylic platform and temperatures were maintained between 37 and 38 C with radiant heat. The skin over the left gluteus muscle was excised, the muscle’s proximal muscle edge was detached from its spinal origin and pinned to a transparent pedestal (Sylgard 184; Dow Corning, Midland, MI, USA). Muscle was superfused throughout the experiment with bicarbonate-buffered physiological salt solution (PSS; NaCl 137 mM, KCl 4.7 mM, MgSO4 1.2 mM, CaCl2 2 mM and NaHCO3 18 mM) which was warmed to 37°C and aerated with 95% N2 and 5% CO2. All reagents were purchased from Sigma-Aldrich Canada (Oakville, Ontario) and Fisher Scientific (Ottawa, ON).

**FIGURE 1:**
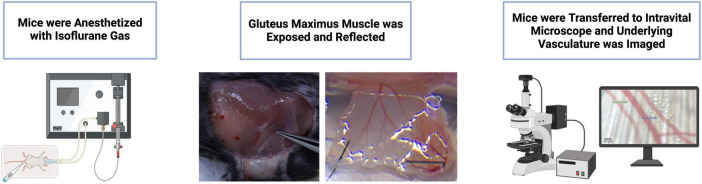
Overview of surgical preparation and vessel visualization using intravital microscopy (IVM). Mice were anesthetized with isoflurane gas, the gluteus maximus muscle was exposed and reflected, and the underlying vasculature was imaged using an intravital microscope. Figure partially created in BioRender.

The preparation was placed on the stage of a modified Nikon Eclipse intravital microscope and equilibrated for approximately 45 min. Arterioles were visualized using bright field microscopy with a Nikon 40X objective lens linked to a Hamamatsu C2400 video camera and Sony PVM-132 video monitor. Arterioles were imaged at a total magnification of 950X. Arteriole internal diameter was evaluated using a video caliper (model 321, Colorado Video Inc, Boulder, CO, USA) with spatial resolution ≤ 1 um. Data collection was performed at 40 Hz using the PowerLab 8S (AD instruments, Castle Hill, Australia) interface. Second-order arterioles were selected for analysis based on their role in regulating blood flow distribution within muscle, with measurements taken from analogous regions across preparations. Baseline diameter was taken at a concentration of 10^–9^ for each reagent tested under control conditions following an equilibration period. Figure 1 provides an overview of the surgical preparation.

### 2.4 Vasoactive agents

Vasoactive chemicals and reagents were sourced from Sigma Aldrich Canada (Oakville, Ontario) or Fisher Scientific (Ottawa, ON). Final working concentrations were obtained through serial dilution in fresh PSS–acetylcholine (ACh; 10-^–9^ to 10^–5^ M; endothelium-dependent vasodilator), phenylephrine (PE; 10^–9^ to 10^–5^ M; α1-adrenoceptor dependent smooth muscle contractor), histamine (His; 10^–9^ to 10^–4^ M; NO-dependent permeability factor), compound 48/80 (10^–9^ to 10^–3^ M; mast cell activator) and sodium nitroprusside (SNP, 10^–3^ M; NO donor).

To assess the impact of the concentrations of vasoactive agents, they were cumulatively added to the superfusion solution in increasing concentration. For each concentration, the arteriolar diameter was stabilized for a minimum of 2 min before the initial measurement and 2 min prior to the administration of subsequent concentrations. Following observations at the peak concentration, the superfusion was reverted to control PSS and the resting diameter was given a recovery period of 15 min before the following agent was assessed. Mice were excluded from the study if they died, or their vasculature was damaged during surgical preparation. Additionally, mice were excluded from analysis of a specific vasoactive agent if data collection was not possible due to a complete lack of response to all concentrations or cessation of blood flow. Since these conditions arose after evaluation of certain agents but not others, the number of mice considered varied for each individual agent tested. Maximum dilation was determined post-superfusion with SNP 10^–3^ M. Those that failed to reach maximum dilation to SNP 10^–3^ M were excluded from analysis of maximum dilation.

### 2.5 Statistical analyses

Statistical analyses were performed using GraphPad Prism 10.0 (GraphPad Prism, Boston, MA, USA).

Data are presented as mean [standard deviation (SD)]. Vessel response was calculated as the percent diameter change, representing the difference between the response and baseline diameter. The baseline diameter was taken at a vasoactive agent concentration of 10^–9^. Vascular responsiveness between WT and Tg mice was analyzed using a two-way ANOVA. The main effect of mouse type (WT vs. Tg) is expressed as a *p*-value superimposed onto relevant figures. The absolute change in vessel diameter (from baseline to maximum) between groups was tested using a two-tailed unpaired *T*-test. Statistical significance is reported using *p < 0.05.

## 3 Results

### 3.1 Animal population

15 WT and 15 Tg mice were studied. 11 WT and 13 Tg mice were considered in at least one aspect of vasoactive response analyses; specific *n*-values for each agent are provided as some mice were included for one agent and excluded for another. For included animals (those with at least one viable response to PE, ACh, His, or compound 48/80), the WT and Tg mice had mean ages (SD) of 34.1 weeks (2.9) and 35.2 weeks (2.9), respectively (*p* = 0.8230). Mean weights were (SD) were 33.48 g (4.00) for WT mice and 28.52 g (3.19) for Tg mice (*p* = 0.0069).

### 3.2 Vasoactive response to phenylephrine

**FIGURE 2:**
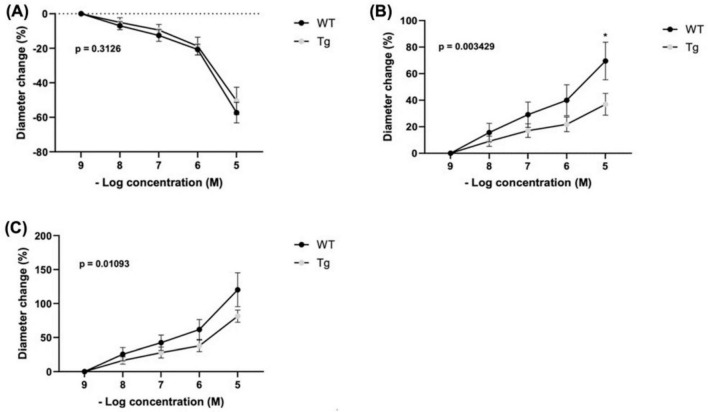
Percent changes in arteriole diameter relative to established baseline diameter (at [10^– 9^ M]) of individual vasoactive agents were measured for **(A)** Phenylephrine (PE) (*n* = 8 WT; *n* = 10 Tg), **(B)** Acetylcholine (ACh) (*n* = 7 WT; *n* = 10 Tg) (* indicates *p* < 0.05) at [10^–5^ M]), and **(C)** Total response (*n* = 5 WT; *n* = 7 Tg) represents the cumulative absolute percent changes in diameter from baseline for ACh and PE at a concentration of 10^– 9^ M for subjects with recordings of both agents. The main effect of mouse type (WT vs. Tg) on percent change in diameter are expressed as superimposed *p*-values.

Changes in arteriole diameter in response to phenylephrine (PE) were assessed relative to the baseline diameter at a PE concentration of 10^–9^ (*n* = 8 WT; *n* = 10 Tg) (Figure 2A). There was a trend toward greater constrictive response among WT compared to Tg mice at higher concentrations, but the differences did not reach statistical significance. The mean percentage changes in diameter for WT vs. Tg mice at each PE concentration were as follows (Figure 2A): 10^–8^: (−6.95 vs. −5.00%), 10^–7^, (−12.55 vs. −9.46%), 10^–6^ (−20.72 vs. −18.75%), and 10^–5^ (−57.29 vs. −50.48%). Further, the main effect of mouse type (Tg vs. WT) on percent diameter change was not statistically significant (*p* = 0.3126).

### 3.3 Vasoactive response to acetylcholine

Arteriole diameter changes were measured relative to baseline diameter at an acetylcholine (ACh) concentration of 10^–9^ (*n* = 7 WT; *n* = 10 Tg) (Figure 2B). Across increasing concentrations, WT mice showed a trend toward greater dilatory response compared with Tg mice. At 10^–8^: (15.59 vs. 9.05%), 10^–7^: (29.10 vs. 17.05%), and 10^–6^: (39.98 vs. 21.83%) the differences were non-significant. However, at a concentration of 10^–5^, WT mice displayed a significantly larger diameter change than Tg (69.55 vs. 36.91%; *p* = 0.0107). Furthermore, two-way ANOVA indicated that mouse type (Tg vs. WT) had a significant main effect on percent change in diameter (*p* = 0.0034).

### 3.4 Total vasoactive responsiveness

The cumulative absolute percent changes in diameter (absolute % change ACh + absolute % change PE, from their respective baselines) (Figure 2C). The analysis included only subjects with both ACh and PE recordings (*n* = 5 WT; *n* = 7 TG). Across four concentrations (10^–8^, 10^–7^, 10^–6^, and 10^–5^), there were no statistically significant differences in cumulative percentage change between WT and Tg individuals, respectively: 10^–8^: (25.66 vs. 16.38%), 10^–7^, (42.40 vs. 27.97%), 10^–6^ (61.77 vs. 37.78%), and 10^–5^ (120.30 vs. 81.41%). However, using two-way ANOVA we found that mouse type (Tg vs. WT) had a significant main effect on percent diameter change (*p* = 0.0109).

### 3.5 Vasoactive response to histamine

**FIGURE 3:**
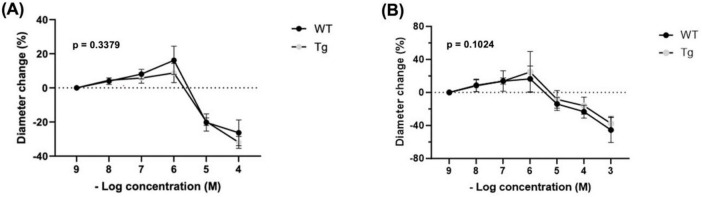
Percent changes in arteriole diameter relative to established baseline diameter (at [10^– 9^ M]) of individual vasoactive agents **(A)** Histamine (*n* = 8 WT, *n* = 8 Tg) and **(B)** Compound 48/80 (*n* = 6 WT, *n* = 6 Tg).

Histamine showed a biphasic response. Percent changes were determined from a baseline diameter at a concentration of 10^–9^ (*n* = 8 WT; *n* = 8 Tg) (Figure 3A). No significant differences were observed at any concentration between WT and TG: 10^–8^: (3.95 vs. 4.48%), 10^–7^: (8.09 vs. 5.79%), 10^–6^: (16.15 vs. 8.74%), 10^–5^: (−20.25 vs. −19.79%), and 10^–4^: (−26.30 vs. −31.92%). Both groups had the highest dilation at a concentration of 10^–6^ and the greatest constriction at 10^–4^. Further, the main effect of mouse type (Tg vs. WT) on percent diameter change was not significant (*p* = 0.3379).

### 3.6 Vasoactive response to compound 48/80

Precent changes were determined from a baseline diameter at a concentration of 10^–9^ (*n* = 8 WT; *n* = 8 Tg) (Figure 3B). No significant differences were observed at any concentrations between WT and Tg mice: 10^–8^ (8.70 vs. 8.06%), 10^–7^ (13.82 vs. 13.84%), 10^–6^ (16.50 vs. 24.93%), 10^–5^ (−13.92 vs. −8.37%), 10^–4^ (−23.27 vs. −16.08%), and 10^–3^ (−45.44 vs. −37.55%). Compound 48/80 showed a biphasic response. Both groups had the highest dilation at 10^–6^ and the greatest constriction at 10^–3^. Further, the main effect of mouse type (Tg vs. WT) on percent diameter change was insignificant (*p* = 0.1024).

### 3.7 Maximum vasodilatory response

**FIGURE 4:**
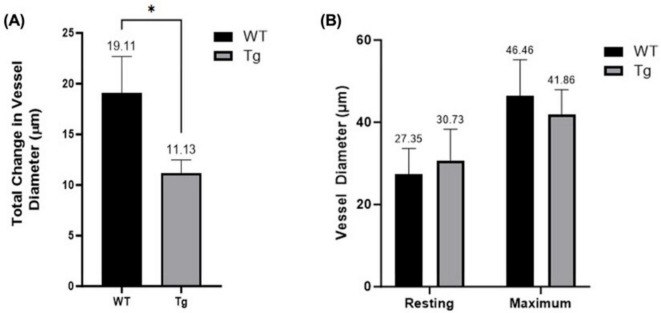
**(A)** Comparison of maximum and resting arteriole diameters in WT and Tg mice (* indicates *p* < 0.05). **(B)** Net diameter change; difference between maximum vessel diameter (following administration of SNP [10^–3^ M]) and resting diameter for both WT (*n* = 10) and Tg (*n* = 12) mice.

Resting diameters were measured at baseline and maximum diameters using SNP 10^–3^. There was a significant difference (*p* = 0.0365) between the mean change from resting to maximum diameter in the WT group (19.11 μm) compared to the Tg group (11.13 μm) (Figure 4A). Mean maximum diameters were 46.46 μm for WT (*n* = 10) and 41.86 μm for Tg (*n* = 12) (*p* = 0.1438) and resting diameters 27.35 μm and 30.73 μm (*p* = 0.2801), respectively (Figure 4B).

## 4 Discussion

This pilot study investigated vascular reactivity in Alzheimer’s disease using IVM in the APP/PS1 (line 85) mouse model (Jackson Laboratories). These mice express the Mo/APP695swe fusion protein and the PS1-dE9 variant, both of which are linked to early-onset familial Alzheimer’s disease and β-amyloid protein production. By employing intravital microscopy, we assessed differences in vasomotor responsiveness of skeletal muscle arterioles to PE, ACh, histamine, and compound 48/80 agents. No significant differences in percent diameter change were observed between WT and Tg mice in response to PE (10^–8^ to 10^–5^), histamine (10^–8^ to 10^–4^), or compound 48/80 (10^–8^ to 10^–3^). Histamine and compound 48/80 exhibited well-documented biphasic responses, with concentrations 10^–8^ to 10^–6^ of histamine and 10^–8^ to 10^–6^ of 48/80 resulting in vasodilation, and concentrations 10^–5^ and 10^–4^ of histamine and 10^–5^ to 10^–3^ of 48/80 resulting in vasoconstriction. Neither dilatory of constrictive responses showed significant differences between WT and Tg populations. However, it appeared vasodilatory capacity of Tg showed a significant reduction in vasodilation at an ACh concentration of 10^–5^ when compared to WT, and there was a significant reduction in net diameter change among Tg mice. Furthermore, there were trends toward better constrictive and total vasoactive response among WT compared to TG mice at higher concentrations, but the differences did not reach statistical significance.

The APP/PS1 (line 85) mouse model mimics AD pathogenesis by promoting elevated β-amyloid peptide production in CNS neurons. Given the bi-directional movement of β-amyloid peptides across the BBB, an increase has been observed in the peripheral blood of APP/PS1 mice (Xiong et al., 2011). β-amyloid peptides have been shown to accumulate in vasculature, triggering inflammation and damage to vascular endothelium and smooth muscle contributing to vascular dysfunction (Anzovino et al., 2023; Koizumi et al., 2015). The findings of the current study align with previous literature showing reduced vasodilatory function in AD (Han et al., 2008; Khalil et al., 2007; Venturelli et al., 2018). Previous studies have suggested reduction in cerebral and peripheral blood flow observed in AD correlates with reduced nitrous oxide (NO) bioavailability (Morris et al., 2014). NO is a critical mediator of ACh-induced vasodilation; and is released from vascular endothelial cells following muscarinic receptor stimulation (Kellogg et al., 2005). However, endothelial-dependent NO release also contributes to the vasodilatory effects of histamine through activation of endothelial H1 receptors (Berra-Romani et al., 2019). Notably, a reduction in vascular responsiveness was not observed between Tg and WT mice with exposure to vasodilatory concentrations of histamine. While mechanisms underlying histamine- and ACh-induced vasodilation involve factors beyond NO, these findings suggest additional factors may be involved in reduced vascular responsiveness to ACh (Han et al., 2008; Khalil et al., 2007; Venturelli et al., 2018). Furthermore, lack of reduction in histamine- and compound 48/80-mediated vasodilation suggests observed reductions in vascular reactivity are potentially mediated more through direct endothelial dysfunction rather than inflammatory processes. Future research should investigate the molecular and morphological changes in vascular structures and their response to vasoactive agents. Such studies could help elucidate the mechanisms underlying AD-associated vascular dysfunction and provide insights into how AD progression affects both peripheral and cerebral vascular function.

Reductions in cerebral and peripheral blood flow have been directly associated with the progression of AD (Venturelli et al., 2018). Reduced blood flow may occur from intravascular accumulation of atherosclerotic or β-amyloid plaques, or from increased vascular resistance or resting vascular diameter. Vascular resistance is governed by the effects of vasodilatory and vasoconstrictive agents and is influenced by not only the concentration of these agents, but also the responsiveness of vasculature. It is feasible that reduced vascular responsiveness may be a potential contributor to reductions in peripheral blood flow observed in AD. This study showed a significant reduction in acetylcholine-mediated vasodilation and reduced net diameter change between Tg and WT mice. Literature has shown that elevated soluble β-amyloid levels can induce both cerebral and peripheral vasoconstriction (Arendash et al., 1999; Suo et al., 1998; Suo et al., 2000). This suggests that increased soluble peripheral β-amyloid levels may contribute to elevated resting vascular tone in AD. However, in the present study, no significant difference in resting vessel diameter was observed between Tg and WT mouse populations. One possibility is that experimental conditions, such as surgical stress and anesthetic use, may have influenced resting tone and vasoreactivity in both groups, potentially minimizing differences in responsiveness.

Despite recent advances, the relationship between peripheral and cerebrovascular dysfunction in AD remains unclear. Dysfunction of peripheral vasculature may indirectly contribute to AD progression through influences on cerebral vasculature. Peripheral vascular disease has been shown to compromise blood-brain barrier integrity through hemodynamic stress and resulting damage. This could impair cerebral β-amyloid clearance, promote cerebral accumulation, vascular deposition, and thereby compromise cerebral vasculature (Nyúl-Tóth et al., 2024; Sagare et al., 2012). Alternatively, peripheral vasculature dysfunction may contribute to sarcopenia and dyanpenia observed in AD. Studies have shown correlations between reductions in muscle mass and strength, and cerebral volume and cognitive decline, in AD (Liu et al., 2022; Ogawa et al., 2018). Peripheral vascular dysfunction could lead to hypoperfusion of skeletal muscle resulting in tissue ischemia, reduced contractility and muscle atrophy. Future research should aim to clarify the temporal relationship between cerebral and peripheral vascular dysfunction and how this relates to AD progression as well as the potential role of peripheral vasculature in motor symptoms observed in AD. These studies could help establish peripheral vasculature as a therapeutic target for the treatment of motor symptoms and possibly cognitive decline. Therefore, peripheral vascular dysfunction may hold utility as a biomarker for AD progression and its treatment could improve the quality of life of AD patients.

This study has limitations. One of the primary constraints is the limited sample size, further compounded by the exclusion of certain mice due to dropout. Furthermore, post-dose-response curve, the Tg group frequently demonstrated a prolonged return to baseline arteriolar diameter, manifesting an element of inconsistency. Enlarging the sample size in subsequent studies could offer greater precision and reduce the degree of inconsistency across experimental responses. It is also worth noting that the experiments and the recording of vessel diameters were conducted by two different observers. This could have introduced measurement bias, potentially amplifying variability in the recorded measurements. However, to minimize this source of variability, we ensured that an equivalent number of WT and Tg mice were assessed by each observer. Finally, alterations in vascular wall morphology and cellular composition, including inward or outward remodeling of arteriole vasculature were not assessed in this study, and could have independently influenced vasoreactivity. This should be assessed in future studies for a greater understanding of the mechanisms involved in reduced vascular responsiveness.

## 5 Conclusion and future directions

This study’s preliminary findings suggest that endothelial dysfunction underlay altered vascular responses in a murine model of AD, evidenced by significant main effects of mouse type on overall responses to acetylcholine and combined vasoactive stimuli (PE + ACh), as well as a notable difference at one ACh concentration (10^–5^) and net change between resting and maximum dilation. Although many differences were not statistically significant, these initial observations lay groundwork for future studies. Moving forward, larger sample sizes, broader age ranges, and additional stressors (e.g., high-fat diets) can provide a more nuanced understanding of how AD and peripheral vasculature interact over time. Employing diverse animal models, combining intravital microscopy with histological analyses, and examining a wider range of vasoactive stimuli may help to elucidate the role of vasomotor dysfunction in AD. The inclusion of female subjects will also help clarify whether sex differences influence peripheral resistance arteries in AD and the impact of hormone differences. By extending the investigation beyond the brain, this research encourages a more integrative view of AD pathogenesis, highlighting the peripheral vasculature’s role and encouraging exploration and development of multimodal therapeutic strategies aimed at vascular targets in the fight against Alzheimer’s disease.

## Data Availability

The raw data supporting the conclusions of this article will be made available by the authors, without undue reservation.
